# Descriptive Study of Headache as the Most Common Presenting Feature of Cerebral Venous Thrombosis

**DOI:** 10.7759/cureus.43007

**Published:** 2023-08-05

**Authors:** Ashutosh K Mishra, Ruchi Shukla, Rameshwar N Chaurasia, Archana Verma

**Affiliations:** 1 Department of Neurology, All India Institute of Medical Sciences, Raebareli, IND; 2 Department of Ophthalmology, All India Institute of Medical Sciences, Raebareli, IND; 3 Department of Neurology, Institute of Medical Sciences, Banaras Hindu University, Varanasi, IND

**Keywords:** neuroimaging, cerebral venous sinus, stroke, headache, cerebral venous thrombosis

## Abstract

Objective: To study the etiology and clinical characteristics of cerebral venous thrombosis (CVT) patients with a detailed description of headache as a presenting feature.

Introduction: CVT is an infrequent type of stroke with protean clinical manifestations. The most common presenting symptom in CVT is headache (>85%), followed by seizures and focal neurological deficits.

Methods: A total of 32 consecutive and confirmed patients of CVT were recruited after obtaining informed consent. CVT was diagnosed based on clinical and imaging parameters. Data regarding etiology, clinical symptoms, and signs with special mention of headache pattern, onset, site, character, severity (based on the visual analog scale), aggravating and relieving factors, as well as sinus involvement were recorded.

Results: A total of 32 patients (16 males and 16 females) with a mean age of 31.56 (SD = 14.31) years were recruited, out of which 31 patients (96.87%) presented with headaches. The mode of onset of headache was acute in 19.35%, sub-acute in 67.75%, and chronic in 12.9% of patients. Location was holocranial in 38.71%, hemicranial in 29.03%, frontal in 22.58%, and occipital in 9.68% of patients. Headache was severe in 38.7% and moderate in 61.3% of patients. Character was throbbing in 67.74%, heaviness in 25.8%, and band-like in 6.46% of patients. Headache was aggravated on bending forward in 58.06%, movement in 35.48%, coughing in 32.26%, straining in 25.8%, and standing in 16.12% of patients. The relieving factors of headache were lying down in 45.16%, sleeping in 45.16%, and sitting quietly in 9.86% of patients.

Conclusion: CVT should be suspected in patients presenting with new-onset holocranial or hemicranial headaches of increasing intensity, thereby requiring early imaging and appropriate management.

## Introduction

Cerebral venous thrombosis (CVT) is a rare form of venous thromboembolism (VTE) [1]. CVT represents almost 0.5-3% of all types of strokes, affecting predominantly younger people [2,3]. CVT occurs in around 10-20% of strokes in young adults [4].

Diagnosis of CVT can be difficult due to its diverse and multiple presentations. Its mode of presentation can be divided into three subtypes, namely, acute, sub-acute, or chronic. Headache is the most common presenting symptom occurring in more than 80% of cases of CVT, followed by seizures in 35-40% of cases [5]. The presentation of headache in CVT is usually acute, non-localized, nonspecific, and dull in character, which is aggravated by maneuvers that lead to rise in intracranial pressure like Valsalva. Occasionally, headaches can also be associated with auras. Headache in CVT can also mimic thunderclap headache, which commonly occurs in subarachnoid hemorrhage [6]. Most often headache is the only manifestation of CVT [7,8].

The aim was to study the etiology and clinical characteristics of CVT patients with a detailed description of headache as a presenting feature.

## Materials and methods

It was a prospective study carried out over a span of two years (June 2017 to May 2019) at a tertiary care hospital in the northern part of India. Diagnosis of CVT was based on clinical features and confirmed on magnetic resonance venography (MRV) as recommended by the European Stroke Organisation guidelines [9]. The continuous sampling method was employed to recruit all the confirmed and consecutive cases of CVT from the outpatient and inpatient departments of neurology. Patients were included after giving informed consent, and ethical approval for this study was granted by the Institute Ethical Committee, Institute of Medical Sciences, Banaras Hindu University, Varanasi (Approval No.: Dean/2016-17/EC/724).

A total of 32 confirmed CVT patients were recruited. A complete history with a full description of the headache with respect to its onset, grades of severity, site, and characteristic pattern along with relieving and aggravating factors was taken. A systemic and neurological examination was performed and the findings were noted in a structured format by the interviewer.

The mode of onset of headache was divided into the following types depending upon the time interval between the initial painful sensation and the most severe pain: acute onset (less than 48 hours), sub-acute onset (48 hours to 30 days), and chronic onset (more than 30 days).

The severity of headaches was measured with the help of a visual analog scale (VAS). In VAS, a numerical pain rating scale of 0-100 was used. The absence of pain was given a score of 0, and the worst pain was given a score of 100. According to the VAS, the patients were divided into severe headache (VAS score > 70), moderate headache (VAS score 30-70), and mild headache (VAS score < 30) groups [10].

The patients excluded from our study were (a) patients not able to give history like unconscious or uncooperative patients, (b) patients who did not give informed consent, and (c) patients with all causes of primary and secondary headaches other than CVT.

Diagnosis of anemia was considered when the hemoglobin level was less than 12 g/dl in women and less than 13 g/dl in men.

Complete ocular examination, including visual acuity, pupillary examination, extraocular movements for cranial nerve palsies, and fundus photograph, was done at the time of presentation as well as in subsequent follow‑up visits. Visual acuity was recorded using the electronic Snellen visual acuity charts and was converted to logMAR. Papilledema was graded clinically based on Friesen’s grading [11].

A detailed history of seizures was taken, and the 2017 International League Against Epilepsy (ILAE) classification was used to categorize seizure subtypes. Glasgow Coma Scale (GCS) and modified Rankin scale (mRS) scores were noted at the time of admission and on subsequent follow-up visits [12].

In all confirmed cases of CVT, a systematic and elaborate etiological investigation was performed, including a complete hemogram, blood sugar levels, kidney and liver function tests, and a complete coagulation profile.

Risk factors and causes of CVT like any hematological disorder, including antinuclear antibody (ANA) and antiphospholipid antibodies (APLA), serum homocysteine, serum protein C, serum protein S, and antithrombin III deficiency, were conducted.

Patients were also tested for local or systemic infections and malignancies with the help of urine routine and microscopic examination, chest X-ray, ultrasonogram (USG) of the whole abdomen, and computed tomography (CT) of the chest and abdomen if required.

All CT and MRV reports were read by expert radiologists. On magnetic resonance imaging (MRI) of the brain with contrast venography (Signa HDxT, GE HealthCare, Chicago, IL), the location and extent of venous sinus occlusion were recorded.

All patients received appropriate treatment with low molecular weight heparin, followed by oral anticoagulants for a minimum period of six months. Patients were also given symptomatic treatment for headaches with analgesics.

Follow-up visits were performed at three months, six months, and 12 months preferably by direct interviews and observations. mRS score was noted at each visit, and MRV was advised at six months and 12 months to look for recanalization. Follow-up patients were divided into different categories as follows: (a) completely recovered: it was defined as the change in mRS score from a baseline of ≥ 2 to an mRS score of 0-1; (b) improved: those patients who showed a reduction in mRS score by 1 or more values but not falling into the criteria for complete recovery were labeled as improved; (c) unchanged; (d) death/deterioration.

Statistical analysis

Descriptive statistics were calculated for all data of sample assays. Analysis of observations was done using SPSS version 19 (IBM Corp., Armonk, NY). A variable with a p-value less than 0.05 was considered to be statistically significant. Demographic data were summarized as mean and standard deviation.

## Results

A total of 32 patients were included. The majority of patients (68.75%) were in the age group of 21-40 years. In this study, the male-to-female ratio was 1:1. Anemia was the major predisposing condition identified in 62.5% of patients. Other predisposing conditions such as hyperhomocysteinemia, alcohol use, oral contraceptive pills use, and protein C deficiency were present in 12.5% of CVT patients, respectively (Table 1).

**Table 1:** Etiology description

Etiology	No. of patients	Percentage
Anemia	20	62.5
Hyperhomocysteinemia	4	12.5
Alcohol	4	12.5
Deep vein thrombosis	1	3.12
Postpartum (N = 16)	4	25
Oral contraceptive pills (N = 16)	2	12.50
Protein C deficiency (N = 8)	1	12.50
Anti-cardiolipin antibodies	1	3.12
Factor V Leiden (N = 6)	1	16.67

Headache was the most common clinical symptom present in 31 (96.87%) patients, followed by nausea in 19 (61.29%) patients (Table 2).

**Table 2:** Distribution of patients according to clinical symptoms

Clinical symptoms	No. of patients	Percentage
Headache	31	96.87
Nausea	19	61.29
Nausea and vomiting	16	51.61
Blurring of vision	13	40.62
Altered sensorium	7	21.87
Fever	4	12.5
Seizures	10	31.25
Generalized	8	25
Complex partial seizure	2	6.25

On observing the presentation of headaches in CVT patients, we found that 19.35% of patients presented with an acute mode of onset, 67.75% of patients presented with sub-acute onset, and 12.90% had a chronic mode of onset of headache.

The most common location of headache was holocranial headache seen in 12 (38.71%) patients followed by hemicranial (29.03%), frontal (22.58%), and occipital (9.68%).

The most common characteristic of headache was throbbing in 21 (67.74%) patients, followed by heaviness in eight (25.80%) patients. Based on the severity of the headache, 12 (38.70%) patients presented with severe headaches, while the majority (19, 61.30%) of patients presented with moderate headaches.

We studied the precipitating factors of headache in which the majority of patients' headache was aggravated by bending forward in 18 (58.06%), followed by movement in 11 (35.48%) and coughing in 10 (32.26%) patients. We also studied the factors that relieved the patient’s headache, among which both lying down and sleeping were found in an equal number of cases, i.e., 14 (45.16%) patients (Table 3).

**Table 3:** Characteristics of headache

Headache characteristics	Number of patients	Percentage
Mode of onset (n = 31)		
Acute	6	19.35
Subacute	21	67.75
Chronic	4	12.90
Location of headache (n = 31)		
Holocranial	12	38.71
Hemicranial	9	29.03
Frontal	7	22.58
Occipital	3	9.68
Character of headache (n = 31)		
Throbbing	21	67.74
Band-like	2	6.46
Heaviness	8	25.80
Intensity of headache (n = 31)		
Moderate (visual analog scale = 30-70)	19	61.30
Severe (visual analog scale >70)	12	38.70
Nature of headache (n = 31)		
Continuous	19	61.30
Episodic	12	38.70
Precipitating factors (n = 31)		
Movement	11	35.48
Straining	8	25.80
Bending forward	18	58.06
Coughing	10	32.26
Standing	5	16.12
Relieving factors (n = 31)		
Lying down	14	45.16
Sleeping	14	45.16
Sitting quietly	3	9.68

Among the clinical signs, 37.5% of patients had papilledema, 12.5% had hemiparesis, while dysarthria and aphasia accounted for 6.25% and 3.12%, respectively (Table 4).

**Table 4:** Distribution of patients based on clinical signs at presentation

Clinical signs	No. of patients	Percentage
1. Focal neurological signs		
(a) Hemiparesis	4	12.5
(b) Dysarthria	2	6.25
(c) Aphasia	1	3.12
2. Papilledema	12	37.5
3. Cranial nerve involvement	3	9.37
4. No signs	10	31.26

Among the sinuses involved in MRV, the sigmoid sinus with or without other sinuses (43.75%) was the most commonly involved sinus (Table 5).

**Table 5:** Distribution of patients based on sinuses involvement

Sinuses involved	No. of patients	Percentage
Superior sagittal sinus with/without other sinuses	12	37.5
Right transverse sinus with/without other sinuses	8	25
Left transverse sinus with/without other sinuses	13	40.63
Sigmoid sinus with/without other sinuses	14	43.75
Straight sinus with/without other sinuses	1	3.12
Isolated superior sagittal sinus	6	18.75
Isolated right transverse sinus	3	9.37
Isolated left transverse sinus	4	12.5
Cortical veins	3	9.37

Parenchymal lesions were identified on MRI in 13 (40.62%) cases, out of which seven patients had hemorrhagic infarction and six patients had non-hemorrhagic infarction (Table 6).

**Table 6:** Distribution of patients based on parenchymal lesions (n = 13)

MRI findings	Number of patients	Percentage
Hemorrhagic infarction	7	53.89
Infarction	6	46.15

The outcome was measured on follow-up on the basis of the mRS score (Tables 7, 8).

**Table 7:** Distribution of patients according to outcome on modified Rankin scale (mRS) score

mRS (score)	Baseline (N = 32)	Outcome at 3 months (N = 32)	Outcome at 6 months (N = 28)
Recovery (0-1)	12 (37.5%)	28 (87.5%)	25 (89.28%)
Functionally independent (2)	12 (37.5%)	3 (9.37%)	3 (10.71%)
Dependent (3-5)	8 (25%)	0	0
Death (6)	0	1 (3.12)	0

**Table 8:** Final outcome according to the modified Rankin scale (mRS)

Outcome	At 3 months (N = 32)	At 6 months (N = 28)
Completely recovered	16 (50%)	14 (50%)
Improved	14 (43.75%)	14 (50%)
Unchanged	1 (3.125%)	_
Deteriorated/death	1 (3.125%)	_

The recanalization of the occluded venous sinuses was observed in MRV on follow-up visits (Table 9).

**Table 9:** Follow-up magnetic resonance venography (MRV) results

On MRV of occluded sinuses	At 6 months (N = 20)	At 12 months (N = 11)
Complete recanalization	8 (40%)	7 (63.63%)
Partial recanalization	9 (45%)	3 (27.27%)
No recanalization	3 (15%)	1 (9.1%)
MRV not performed	8	4

## Discussion

Numerous risk factors and etiologies have been associated with CVT in previous studies. In the present study, a high percentage of patients (62.50%) had anemia at presentation. Whereas in a study by Coutinho et al., anemia was reported in 27% of cases [13]. This discrepancy can be a reflection of a high incidence of anemia in the Indian population, particularly in pregnant females. Therefore, anemia as an independent risk factor of CVT needs further evaluation.

The clinical findings in CVT are explained by two different mechanisms: (a) venous ischemia/infarction or hemorrhage leading to focal brain injury and (b) impaired venous drainage leading rise in intracranial pressure (ICP).

The earliest and the most frequent presentation of CVT is a headache, which occurred in 98.6% of patients in our study. These results were consistent with a study done by Ferro et al. and the Nizam's Institute Venous Stroke Registry (NIVSR) cohort, in which 92% and 83.3% of patients presented with headaches, respectively [14,15].

The main mechanisms postulated in the causation of headache are (a) nerve fiber stretching in the wall of the occluded sinus and (b) the dilatation of blood vessels leading to the local inflammatory reaction, which is seen as an empty delta sign on contrast-enhanced CT.

Other factors which may lead to headaches in CVT are raised ICP, sulcal subarachnoid blood, intracerebral lesions, and meningitis [16].

In this study, the mode of onset of headache was mostly sub-acute in 67.75% of patients. The mode of onset was similar to a study done by Damak et al., in which sub-acute onset was seen in 72% of patients [17]. In terms of the character of headache in our study, 67.74% of patients with CVT presented with throbbing quality. In a study by Botta et al., 44.7% of patients presented with a throbbing type of headache and 4.3% with both heaviness and band-like headache [18]. The results of this study were somewhat similar to our study. In a study by Cumurciuc et al., the headache was throbbing in character in 76% of patients [7]. The throbbing quality of a headache can be explained by the stretching of perivascular nerves [19].

With respect to the site, the headache was mostly holocranial (38.71%), followed by hemicranial (29.03%), frontal (22.58%), and occipital (9.68%). Our findings were similar to that of Botta et al., in which headache was holocranial in 36.2%, occipital in 8.5%, and frontal in 27.7% of patients [18], but different from the study by Wasay et al., in which most of the patients had unilateral headache [20]. The reason behind the difference in the laterality of headaches could be due to the involvement of the sinuses. Most of the patients in our study had involvement of multiple sinuses in which the pathogenesis of headache is due to the rise in ICP leading to diffuse stretching of dura mater, thereby resulting in nonlocalized holocranial headache. Also, many patients had involvement of superior sagittal sinus, which being in midline gives rise to holocranial or poorly localized headache. The pathogenesis of unilateral headache has been postulated to be stretching of the dura of the ipsilateral sinus, which sends painful sensations through the trigeminal nerve [18].

Headache was precipitated by bending forward in 58.06%, head movement in 35.48%, coughing in 32.25%, straining in 25.80%, and standing in 16.12% of patients. In a study done by Botta et al., the aggravating factors of headache were coughing in 46.81%, head movement in 61.7%, standing up in 42.6%, straining in 59.57%, and bending forward in 53.19% of patients [18]. These results were almost comparable to our study results.

Relieving factors in our study were lying down in 45.16%, sleeping in 45.16%, and sitting quietly in 9.68% of patients. This was similar to the study by Botta et al., in which relieving factors of headache were sleeping in 31.9%, lying down in 38.3%, and sitting in 14.9% of patients [18].

On MRV in our study, isolated single sinuses were less commonly involved as compared to the involvement of multiple sinuses together. The sigmoid sinus with or without other sinuses was involved in 43.75% of cases, followed by the left transverse sinus with or without other sinuses (40.63%), superior sagittal sinus with or without other sinuses (37.50%), and right transverse sinus with or without other sinuses (25.0%). The reason for the higher percentage of sigmoid sinus involvement can be its association with the involvement of the right and left transverse sinus.

In earlier studies, including ours, a good clinico-radiological correlation with respect to headache could not be established, and the reason for this could be the rare involvement of isolated venous sinuses.

Most of the patients had recovery on mRS score at three months, i.e., 87.5%. These results were similar to a study done by Singh et al., who had good/independent outcomes on mRS in 60% of patients at discharge and in 82.5% at one year [21].

There has been a long-term debate on whether the decision regarding duration of anticoagulant therapy should be based on recanalization of involved sinuses but a clear consensus has not been reached. Complete recanalization was achieved in about two-thirds of the cases at 12 months. Other studies like that of Baumgartner et al. have shown recanalization in most patients at four months while in a study by Aguiar et al., the recanalization was as high as 85% [22,23].

The major limitation of the study was the small sample size, which might have led to differences in the results from the previously published literature. Hence, recruiting a larger number of participants in further study will enlighten us more on this rare form of stroke.

## Conclusions

This study reaffirms headache to be the most common presenting feature of CVT, followed by blurred vision, seizures, focal neurological deficit, and altered sensorium. CVT is a rare but treatable cause of stroke; therefore, we should have a high index of suspicion for CVT in young patients presenting with acute onset intractable headaches. CT scan of the head is normal in nearly half of the patients; therefore, MRV should be the diagnostic modality of choice.
